# Whole-Genome Sequences of Three Symbiotic *Endozoicomonas* Bacteria

**DOI:** 10.1128/genomeA.00802-14

**Published:** 2014-08-14

**Authors:** Matthew J. Neave, Craig T. Michell, Amy Apprill, Christian R. Voolstra

**Affiliations:** a Red Sea Research Center, King Abdullah University of Science and Technology, Thuwal, Saudi Arabia; b Woods Hole Oceanographic Institution, Woods Hole, Massachusetts, USA

## Abstract

Members of the genus *Endozoicomonas* associate with a wide range of marine organisms. Here, we report on the whole-genome sequencing, assembly, and annotation of three *Endozoicomonas* type strains. These data will assist in exploring interactions between *Endozoicomonas* organisms and their hosts, and it will aid in the assembly of genomes from uncultivated *Endozoicomonas* spp.

## GENOME ANNOUNCEMENT

*Endozoicomonas* spp. (*Gammaproteobacteria*; *Oceanospirillales*) are dominant members of the bacterial community associated with diverse marine invertebrates, including corals (1–6), sponges (7), gorgonians (8, 9), molluscs (10–13), and tubeworms (14), as well as a basal chordate (15). In some hosts, these bacteria have been observed intracellularly (2, 11, 13). However, despite the apparent importance of *Endozoicomonas* spp., it is not clear how they interact with their host. For example, they are the dominant bacteria in seemingly healthy animals (3, 8, 15, 16), although they have been implicated as the causal agent of disease in fish (17). Clarifying the functional capabilities of *Endozoicomonas* has been challenging because they reside in or on a host organism and can be difficult to culture (6). Only a handful of isolates are available in culture collections (7, 10, 12, 18, 19). Thus, metagenomic or single-cell analyses may be useful techniques for assessing the genomic capabilities of these bacteria; however, a lack of genetic resources hampers these approaches. To address this issue, we sequenced the genomes of three publically available *Endozoicomonas* type strains.

*Endozoicomonas elysicola* DSM 22380 (12) and *Endozoicomonas numazuensis* DSM 25634 (7) were obtained from the German Collection of Microorganisms and Cell Cultures (DSMZ) (Braunschweig, Germany), and *Endozoicomonas montiporae* LMG 24815 (19) was obtained from the Belgian Coordinated Collections of Microorganisms (BCCM) (Ghent, Belgium). We prepared small-insert libraries by shearing isolated DNA into 180-bp fragments and processing with the NEBNext library preparation kit (New England BioLabs). Long libraries averaging approximately 2 kb were prepared according to the Nextera mate-pair sample preparation kit (Illumina). The small-insert libraries were sequenced using the Illumina HiSeq platform (100-bp paired-end reads), and the long mate-pair libraries were sequenced using the Illumina MiSeq platform (150-bp paired-end reads). Approximately 10 million paired-end reads were obtained for each library and each *Endozoicomonas* strain.

The small-insert reads were trimmed for quality, and the Illumina adapters were removed using Trimmomatic (20). Fragments with both surviving read pairs were then digitally normalized using the recommended protocol in khmer (21–23). The long mate-pair reads were trimmed using NextClip (24), and fragments with the junction adapter in at least one of the paired reads were used in the assembly. The small- and long-insert libraries were error corrected and assembled using the AllPaths-LG assembler (25), and the gaps in the resulting scaffolds were closed using GapFiller (26). A small number of scaffolds were further joined after a manual examination of mate-pair mappings using Circos (27). The genomes assembled into ≤31 scaffolds, with a scaffold *N*_50_ of >0.92 Mbp (Table 1). The whole-genome sequences were annotated using the NCBI Prokaryotic Genome Annotation Pipeline (http://www.ncbi.nlm.nih.gov/genome/annotation_prok/).

**TABLE 1:** Sequencing and assembly results for the *Endozoicomonas* type strains

Strain	Species	Host organism	GenBank accession no.	Genome size (Mbp)	No. of scaffolds	Scaffold *N*_*50*_ (Mbp)	No. of contigs	No. of ORFs^*a*^	No. of 5S rRNAs	No. of 16S rRNAs	No. of 23S rRNAs	G+C content (%)
DSM 22380	*E. elysicola*	Sea slug, *Elysia ornata*	JOJP00000000	5.61	2	5.57	21	4,270	8	6	6	46.8
LMG 24815	*E. montiporae*	Coral, *Montipora aequituberculata*	JOKG00000000	5.60	20	1.02	83	4,362	8	4	4	48.5
DSM 25634	*E. numazuensis*	Sponge, cf. *Haliclona* spp.	JOKH00000000	6.34	31	0.92	131	4,650	3	5	2	47.1

a ORF, open reading frame.

The *Endozoicomonas* genomes were large (>5 Mbp; Table 1) and contained versatile metabolic strategies, including the complete Embden-Meyerhof-Parnas glycolytic pathway, the complete tricarboxylic acid cycle, and genes for the conversion and assimilation of nitrate. Although a genome sequence of *E. elysicola* was already available (28), we provide ordered contigs in an almost-closed scaffold for gene synteny studies, which was not available previously.

### Nucleotide sequence accession numbers.

These whole-genome shotgun projects have been deposited at DDBJ/EMBL/GenBank under the accession numbers given in Table 1. The versions described in this paper are the first versions.
